# SPECT and PET myocardial perfusion imaging in Austria, Germany, and Switzerland results of the first joint survey of 2021

**DOI:** 10.1007/s00259-023-06336-8

**Published:** 2023-07-15

**Authors:** O. Lindner, M. Hacker, W. Burchert, R. R. Buechel

**Affiliations:** 1 Institut für Radiologie, Nuklearmedizin und Molekulare Bildgebung, Herz- und Diabeteszentrum NRW, Georgstr. 11, Bad Oeynhausen, Germany; 2 Klinische Abteilung für Nuklearmedizin, Universitätsklinik für Radiologie und Nuklearmedizin, Medizinische Universität Wien, Vienna, Austria; 3 Klinik für Nuklearmedizin, Universitätsspital Zürich, Zurich, Switzerland

**Keywords:** Myocardial perfusion scintigraphy, Utilisation review, Utilisation statistics, Numerical data, Amyloidosis imaging

## Abstract

**Purpose:**

This paper presents the results of the first joint survey on the use of SPECT and PET myocardial perfusion imaging (MPI) and cardiac amyloidosis imaging in Austria, Germany, and Switzerland of the year 2021.

**Methods:**

A questionnaire was sent in 2022 to centres practicing nuclear medicine.

**Results:**

Data from 14 Austrian (10,710 SPECT), 218 German (133,047 SPECT), and 16 Swiss centres (11,601 MPI (6,879 SPECT, 4722 PET)) were analysed. In Austria and Germany, the PET MPI numbers were close to zero and not considered. Official MPS numbers from 2015 to 2021 from Austria and Germany revealed a decline in Austria by about 40% in the pandemic years 2020 to 2021, but an increase in Germany by 9%. Ambulatory care cardiologists represented the major referral group (56–71%). Mostly, stress tests were performed pharmacologically (58–92%). Contrary to Germany, a 1-day protocol was predominant (58–97%) in Austria and Switzerland. The leading camera systems were SPECT-CT in Austria and Switzerland (57–79%) and multi-head systems in Germany (58%). Switzerland had the highest proportion of SPECT MPI with attenuation correction (84%), followed by Austria (43%), and Germany (33%). Electrocardiogram-gated SPECT MPI showed an overall high penetration of 87–99%. Scoring was most frequently applied in Germany (72%), followed by Austria (64%), and Switzerland (60%). Related to the population, the number of cardiac amyloidosis imaging was highest in Austria, followed by Switzerland and Germany.

**Conclusions:**

This first joint survey of 2021 shows considerable differences among the countries. The Swiss situation is outstanding due to the wide use of PET MPI. In terms of camera equipment, Switzerland is also leading, followed by Austria and Germany. Despite the differences in procedural issues, the results reveal an overall high standard of MPI imaging.

## Introduction

Cardiac imaging is an essential element in the diagnostic pathway for chronic coronary syndrome (CCS). SPECT and PET myocardial perfusion imaging (MPI), stress echocardiography, and cardiac magnetic resonance are available as functional non-invasive tests, and cardiac CT is a morphological non-invasive test. Details on the utilisation of these tests throughout Europe are rare. The last published European data refer to the years 2007 and 2016 [1, 2].

In Germany, a regular survey on MPI, including technique, utilisation, and development, has been performed since 2006. Compiled data from 2012 to 2021 have been published recently [3].

Against this background, the idea arose to extend the German survey of the reporting year 2021 to Austria and Switzerland. Due to the increasing application of bone scintigraphy for cardiac amyloidosis imaging, this procedure was also taken into account. The current paper presents the data from the three countries. Although the German data are already available, they are listed again for better comparability [4].

## Methods

In Germany, the updated database from the 2018 survey was used to contact departments and physicians practicing nuclear medicine.

In Austria, departments performing MPI were identified with the help of the Austrian Society of Nuclear Medicine and Molecular Imaging (OGNMB) supplemented by addresses from the German hospital address book [5]. In addition, all responding departments were contacted again in February 2023 and asked to provide their examination numbers for PET MPI along with the radiopharmaceutical used.

In Switzerland, departments performing MPI were identified with the help of the Swiss Society of Nuclear Medicine (SGNM). All departments were directly contacted.

The German one-page questionnaire with a cover letter was sent by fax or email in the first quarter of 2022. In case of no feedback, a reminder was sent about 4 weeks later, and in some cases after personal contact. The survey closed at the end of May 2022.

The questions to be answered in all countries were as follows:

- number of patients examined,
- number of stress and rest procedures,
- number of different types of stress tests,
- number of patients by study protocol,
- percentage of patients examined with gated SPECT,
- percentage of patients examined with attenuation correction (AC),
- type of attenuation correction,
- usage of semiquantitative scoring (categories: never, always, intermediate (= between “never” and “always”)),
- type of camera,
- percentage referrals from cardiologists, primary care physicians, from in-patient ward physicians, and others,
- number of bone scans for diagnosing cardiac amyloidosis and estimated positive rate.

To verify the representativeness of the survey and to reliably estimate the total of SPECT MPI numbers in Germany in 2021, the survey figures obtained were related to the data of the National Association of Statutory Health Insurance Physicians (NASHIP) (Kassenärztliche Bundesvereinigung (KBV; www.kbv.de)) as described earlier [3].

In Austria, official SPECT MPI numbers were delivered by the Austrian National Public Health Institute (GÖG; www.goeg.at) under the supervision of the Federal Ministry of Social Affairs, Health, Long-Term Care and Consumer Protection.

In Switzerland, no official data are readily available. However, only 3 departments (one small private institution, one small, and one medium-sized hospital) did not answer. It can be assumed that > 90% of all patients examined were covered in Switzerland.

Furthermore, the SPECT MPI numbers were related to the invasive coronary angiography (ICA) data of the most recent German cardiology report, which refers to the year 2020 [6]. The Swiss [7] and the Austrian ICA numbers refer to the year 2021, the latter provided by GÖG.

## Results

### Examination numbers and regional distribution

Table 1 lists the numbers of MPI patients recorded in the surveys, their statistical characteristics, the ICA, and the ICA/MPI ratio. Both in Austria and in Germany, nearly no PET MPI was performed. Therefore, no data is listed.

**Table 1:** MPI procedures in 2021

	Austria	Germany	Switzerland		
	SPECT	SPECT	SPECT	PET	Sum of SPECT and PET
Number of centres	14	218	16	6	16
Patients in survey	10,710	133,057	6879	4722	11,601
Total MPI patients*	27,030	246,402	-	-	-
% total patients	39	54	-	-	-
MPI/100,000 population*	292	303	-	-	-
Mean/centre	765	610	430	787	725
ICA	58,889	798,751			30,042
ICA/100,000 population	636	982			344
ICA/MPI	2.2	3.2			2.6**

*MPI*, myocardial perfusion imaging; *ICA*, invasive coronary angiography

*Austria: data according to the Austrian National Public Health Institute (www.goeg.at); Germany: estimate based on the statistics of NASHIP (National Association of Statutory Health Insurance Physicians)

**Since the total Swiss numbers of SPECT and PET MPI are missing, the rate refers only to the number submitted in the survey. The “true” rate is probably only slightly lower as > 90% of all examinations were recorded in the survey

**Fig. 1:**
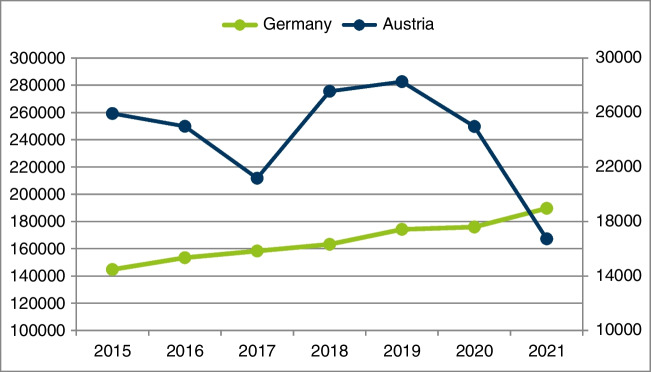
Official SPECT MPI numbers in Austria and Germany from 2015 until 2021. Left-hand scale: Germany; right-hand scale: Austria

Figure 1 shows the time course of the official data in Austria and Germany from 2015 on. As of this year, data from both countries are available. Data from Switzerland are missing. For Austria, the numbers of the code DA010 (myocardial scintigraphy) of the Austrian benefit catalogue are given; for Germany, the numbers of the fee schedule item 17330 (stress SPECT MPI) are given.

### Referrer structure

**Fig. 2:**
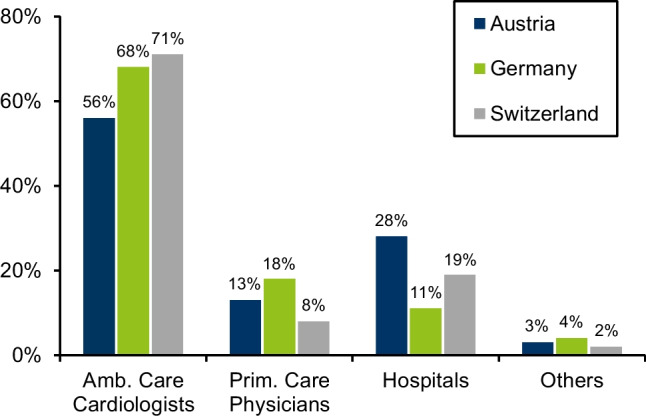
Referral structure in 2021

Figure 2 shows the referrer structure for SPECT and PET MPI. The pattern is roughly similar in all countries. Ambulatory care cardiologists represented the major referral group (> 50%). In Austria, the hospital proportion was the highest.

### Stress tests

**Fig. 3:**
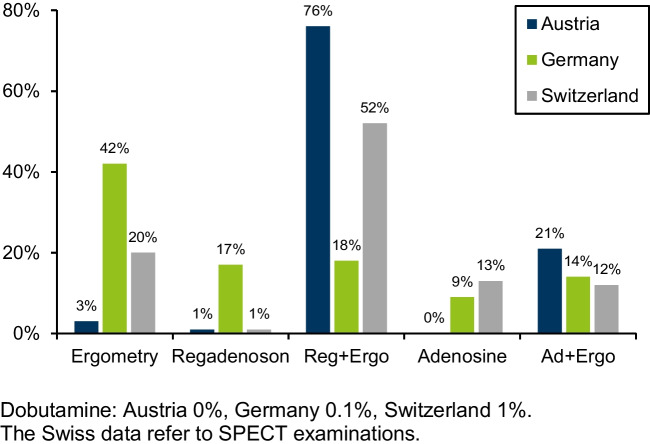
Stress tests in 2021. Dobutamine: Austria 0%, Germany 0.1%, Switzerland 1%. The Swiss data refer to SPECT examinations

The use of the different stress modalities and agents is depicted in Fig. 3.

Most stress tests were performed pharmacologically in all countries. Regadenoson was the preferred stress agent. The proportion of adenosine was similar in all countries. In Austria, nearly all pharmacological stress tests were combined with ergometry, followed by Switzerland and Germany. Dobutamine played no role.

Ergometry only had the highest share in Germany, and it played almost no role in Austria.

### Protocols

**Fig. 4:**
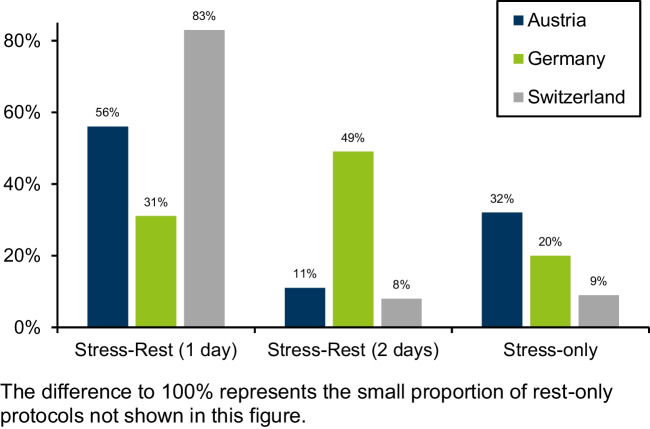
Protocols in 2021. The difference of 100% represents the small proportion of rest-only protocols not shown in this figure

The use of the different SPECT and PET MPI study protocols is shown in Fig. 4. No department applied for Tl-201 exclusively for SPECT MPI.

In Austria and Switzerland, the 1-day protocol was predominant; the 2-day protocol had only a small proportion. The German situation was vice versa.

Stress-only imaging was most frequently performed in Austria, in Switzerland in less than 10%, and in Germany in between.

Rest-only protocols were used very rarely (< 1%).

### Camera systems

**Fig. 5:**
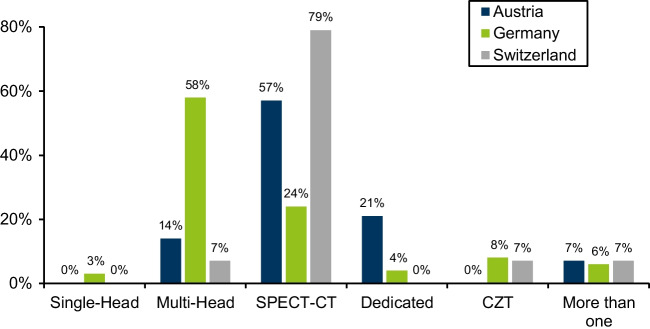
Camera systems by centres in 2021

The camera systems used for SPECT MPI are depicted in Fig. 5.

Single-head cameras were still used to a very limited extent in German centres but no longer in Austria or Switzerland. The leading camera system in Austria and Switzerland was SPECT-CT, with the highest proportion in Swiss centres. In Germany, these systems were used to a much lesser extent.

Multi-head cameras were the main system for MPI imaging in Germany. In Austria and Switzerland, their share was significantly lower.

Dedicated cardiac camera systems were mostly represented in Austria, whereas CZT systems were only used in Germany and Switzerland.

More than one camera system was available in about 7% of the centres in the countries.

### Attenuation correction

Switzerland was the country with the highest proportion of SPECT MPI with AC in terms of number of patients and of centres (Table 2). If the 4722 patients examined with PET were added, the proportion of patients with AC rose to 84%.

**Table 2:** Attenuation correction in MPI in 2021

	Austria	Germany	Switzerland
Prone/supine imaging	0%	8%	0%
Transmission sources	0%	1%	0%
CT-based AC	57%	30%	81%
> one AC method available	0%	1%	0%
No attenuation correction	43%	60%	19%
Patients studied with AC	43%	33%	72%

The values in the upper 5 lines refer to the number of responding centres

In Austria and Switzerland, CT-based AC was the only method applied.

### Gated SPECT and scoring

Data are listed in Tables 3 and 4. With respect to MPI performed as gated SPECT, all countries showed a high penetration with near-perfect values in Switzerland. Interestingly, rest imaging as gated SPECT had a considerably lower share than gated stress in Austria and differed significantly in this point from Germany and Switzerland.

**Table 3:** MPI performed as gated SPECT in 2021

	Austria	Germany	Switzerland
Gated stress	87%	89%	99%
Gated rest	61%	88%	99%
Gated both	60%	87%	99%

Data represent percentages of MPI performed as gated SPECT

**Table 4:** Utilisation of perfusion scores in 2021

	Austria	Germany	Switzerland
Regular	64%	72%	60%
Intermediate	22%	15%	33%
Never	14%	13%	7%

Data represent percentages of centres

Scoring on a regular basis was most frequently applied in Germany. The values for Austria and Switzerland were lower but of the same order of magnitude. The proportion of centres not scoring was lowest in Switzerland.

### Imaging for cardiac amyloidosis

The number of scintigraphies and/or SPECT imaging with bone tracers for suspected or known cardiac amyloidosis, the proportion per 100,000 inhabitants, and the estimated positive rate are given in Table 5. The latter was about the same order of magnitude in the three countries.

**Table 5:** Imaging for cardiac amyloidosis in 2021

	Austria	Germany	Switzerland
No. of patients	919	2947	524
No/100,000 inhabitants	10.2	3.5	6.0
% positive	29%	37%	37%

## Discussion

This paper presents the results of the first joint survey on SPECT and PET MPI in Austria, Germany, and Switzerland, which refers to the year 2021. The German long-term data have been published recently [3]. For a better understanding of the results, it must be considered that the countries have different framework conditions in their health sectors. For example, there is reimbursement for cardiac PET MPI in Switzerland. In Austria, nuclear medicine (SPECT and PET) is centralized within hospitals. In Germany, private practices providing ambulatory care mostly perform SPECT (in 2021, 70% of all MPI [4], hospitals perform SPECT and PET). In both countries, PET systems are nearly solely used for oncological indications. There is no cardiac PET reimbursement by the statutory health insurance.

This point explains why there are such large differences in the use of PET MPI between Switzerland and Austria/Germany. In both latter countries, cardiac PET is only performed in very few centres. Exact figures are not available but are certainly very low.

Another interesting issue is the course of SPECT MPI numbers in Austria and Germany in recent years. In Germany, an increase in examinations has been observed for many years. The COVID-19 pandemic had no real influence on this development [3]. In Austria, on the other hand, SPECT MPI numbers decreased in the pandemic years by 40% (!). As most of the investigations are performed in hospitals, this might be due to the restricted access of outpatients to hospitals during this period. These results are in line with numbers reported by other studies [8–10].

A look at the overall figures shows that Germany and Austria do not differ in terms of examinations per 100,000 inhabitants. At this point, a comparison with Switzerland is not possible due to missing official total numbers.

With about three ICA per MPI, Germany has the highest (most unfavourable) ratio of the three countries and the highest number of angiographies per 100,000 population. From the MPI view, the situation is more favourable in Austria and Switzerland. However, the Swiss ratio in Table 2 is based on the survey data alone. The “true” MPI numbers are likely to be a little bit higher and thus the ratio is somewhat lower.

All other imaging procedures are not likely to have a significant impact on the ICA/MPI ratio [3].

In Germany, a centralisation of investigations has become apparent over the years, which results in an average number of SPECT MPI of 610 per centre. In Austria with 765 MPI studies and Switzerland with 725 studies per centre, the average is even higher. These data are encouraging because expertise runs parallel with the number of studies and is an indirect indicator of good quality.

The referrer structure is roughly the same in all countries. According to the diagnostic decision pathway, most referrals come from cardiology [11]. In Austria and Switzerland, the proportion of hospitals is higher than in Germany. At least in Germany, this is probably due to the shift of examinations to the outpatient sector.

Stress testing clearly shows a shift to pharmacological stress mostly with Regadenoson combined with ergometry. Ergometry alone is still regularly applied in Germany. However, the time course shows a decreasing proportion over the years [3].

There are considerable differences between the three countries in the protocols used. In Austria and Switzerland, 1-day protocols are mostly applied, while in Germany, 2-day protocols with a lower radiation dose to patients are preferred. In Austria, the number of stress-only examinations is the highest. Assuming a similar distribution of patients undergoing MPI for CCS in these countries, the resulting proportions should be comparable.

The camera systems for SPECT MPI are at a high level. Switzerland shows an outstanding situation with 79% of centres equipped with SPECT-CT cameras and with the highest proportion of SPECT patients studied with AC. In Germany, multi-head cameras dominate. The German surveys have shown that a slow but steady transition to SPECT-CT systems is underway [3]. Austria is in between and reaches, with SPECT-CT systems and dedicated cameras, an excellent proportion of 78%. CZT cameras are used in Germany and Switzerland in a small amount. The first systems were installed in Austria in 2022, which, however, was not yet reflected in the presented numbers from 2021.

Attenuation correction is nearly completely based on CT. In Germany, prone-supine imaging is also used as an alternative approach to attenuation correction in a few centres.

Gated SPECT shows high penetration in all countries, with ideal numbers in Switzerland. Interestingly, in Austria, gated SPECT is performed less frequently in rest examinations than in Germany or Switzerland. The reasons for this are unknown. It should be considered that gated SPECT in both stress and rest studies delivers information on ischemia-induced transient ventricular dysfunction (stunning). Especially gated SPECT of the rest study can be used for the identification of absorption artefacts.

Scoring reveals some differences among the countries. Austria and Switzerland are at a comparable level. Germany has the highest share of regular scoring. It remains unclear what the differences are based on. The scoring issue appears to be an objective of continuing education.

### Cardiac amyloidosis imaging

The number of scintigraphies and/or SPECT imaging for cardiac amyloidosis is relatively low compared to the number of MPI studies. However, new therapeutic options have made this imaging an element in the diagnostic process [12].

Related to the population of the countries, the number of amyloidosis scans is highest in Austria, followed by Switzerland and Germany. A positive rate of about one-third can be regarded as sufficient pre-selection of patients. The higher Austrian number of examinations per 100,000 inhabitants and the lower positive rate compared to the other countries indicate a broader application of cardiac amyloidosis imaging.

## Conclusion

This first joint survey of 2021 shows considerable differences among the countries. The Swiss situation is outstanding due to the wide use of PET MPI. In terms of camera equipment, Switzerland is also leading, followed by Austria and Germany. Despite differences in procedural issues, the results reveal an overall high standard of MPI imaging.

Further surveys beyond this one single effort are necessary to gain an overview of further development.

## Data Availability

The data on which this article is based are not publicly available in order to protect the privacy of the departments submitting their data. This was explicitly promised to all participants. Data can be passed on anonymously by the corresponding author on reasonable request.
